# Understanding the cellular uptake and genotoxic potential of industrial relevant nanomaterials utilizing electron microscopy and the ToxTracker assay *in vitro*

**DOI:** 10.1093/mutage/geaf013

**Published:** 2025-07-17

**Authors:** Stephen J Evans, Nynke Moelijker, Inger Brandsma, Michael J Burgum, Rosalie Elespuru, Giel Hendriks, Shareen H Doak

**Affiliations:** In Vitro Toxicology Group, Institute of Life Science, Swansea University Medical School, Swansea University, Singleton Park, Swansea SA2 8PP, Wales, United Kingdom; Toxys B.V., Leiden Bio Science Park, De Limes 7, 3042 DH Oegstgeest, The Netherlands; Toxys B.V., Leiden Bio Science Park, De Limes 7, 3042 DH Oegstgeest, The Netherlands; In Vitro Toxicology Group, Institute of Life Science, Swansea University Medical School, Swansea University, Singleton Park, Swansea SA2 8PP, Wales, United Kingdom; 3741 Thomas Point Road, Annapolis, MD 21403, United States; Toxys B.V., Leiden Bio Science Park, De Limes 7, 3042 DH Oegstgeest, The Netherlands; In Vitro Toxicology Group, Institute of Life Science, Swansea University Medical School, Swansea University, Singleton Park, Swansea SA2 8PP, Wales, United Kingdom

**Keywords:** nanomaterial, genotoxicity, ToxTracker, uptake

## Abstract

Evaluating the genotoxic potential of nanomaterials (NMs) presents unique challenges not associated with traditional toxicological assessment. A key question in any NM focused toxicity study is whether the material has reached the target cell and what its subsequent subcellular localization is. This current study aimed to assess the potential of a panel of industrially relevant NMs; TiO_2_-NM102, TiO_2_-NM105, TiO_2_-E171, silica, polyethylene, polystyrene, carbon black, gold nanorods, tungsten carbide/cobalt, and tungsten carbide, to undergo cellular uptake in mouse embryonic stem cells, which are applied in the ToxTracker genotoxicity assay. Ultrastructural cellular analysis by transmission electron microscopy was undertaken following 100 μg/ml treatment with the test NMs for 24 h; any observed uptake was confirmed by energy-dispersive X-ray spectroscopy. Induction of DNA damage, cytotoxicity, p53 activation, protein stress, and oxidative stress was evaluated by the ToxTracker assay following 24-h treatment with the test NMs (0−100 μg/ml) in the absence of S9. TiO_2_-NM105, silica, polystyrene, carbon black, and tungsten carbide were all shown to undergo cellular uptake, localized in membrane-bound vesicles within the cytoplasm. None of the internalized NMs promoted a genotoxic response in ToxTracker, and similarly, no DNA damage was observed by the materials not internalized. Interestingly, of the internalized NMs, only polystyrene caused a slight cytotoxic response at 100 μg/ml treatment (10% loss in cell viability). Of the NMs not internalized, cytotoxicity was observed in mES cells treated with 100 μg/ml TiO_2_-NM102 (15%), polyethylene (15%), gold nanorods (35%), and tungsten carbide/cobalt (45%). In summary, this study demonstrated that TiO_2_-NM105, silica, polystyrene, carbon black, and tungsten carbide are non-genotoxic *in vitro* despite undergoing cell uptake in the ToxTracker cells. A continued focus is needed to supplement NM genotoxicity studies with cellular uptake analysis.

## Introduction

Nanomaterials (NMs) are being developed for a wide variety of applications in many sectors, including the chemical, pharmaceutical, and food industries. These substances with exceptional properties provide opportunities for product improvement and the development of completely new materials. However, evaluating the genotoxic potential of NMs presents unique challenges not associated with traditional pharmaceutical and chemical toxicological assessment [1]. A NM’s extra- and inter-cellular interactions will be determined by its physico-chemical properties, including size, shape, surface charge/chemistry, and agglomeration state [2]. These properties will influence interactions at the cell membrane, facilitating or hindering cellular uptake. This is especially relevant for non-soluble NMs due to their potentially high bio-reactivity [3, 4].

Previously a working group, the Genetic Toxicology Technical Committee of the International Life Sciences Institute’s Health and Environmental Sciences Institute made recommendations for how NM genotoxicity evaluation should be undertaken [5]. The group recommended a revised genotoxicity test battery for NMs that includes *in vitro* mammalian cell mutagenicity and clastogenicity assessments; *in vivo* assessments would be added only if warranted by information on specific organ exposure or sequestration of NMs. Furthermore, verification of cellular uptake into target cells should be undertaken, alongside *in situ* material physico-chemical characterization prior to endpoint toxicological analysis. Uptake analysis of NMs in the cells of a test system provides vital information that can be relevant to their mode of action if DNA damage is induced [6]. For example, if NMs can theoretically enter the nucleus, they may have the opportunity to directly interact with DNA [7], whilst localization of the test material within the cytoplasm alongside a positive DNA damage response would imply the induction of indirect genotoxicity, such as oxidative stress or protein function inhibition [8, 9].

Identification of a test material within specific organelles or membrane-bound vesicles can also provide explanatory information (when associated with other mechanistic analysis). For example, lysosomes are the most acidic cellular organelle, with an optimal pH 4.5–5.5; NMs within these organelles may be susceptible to acid-denaturation and enzymatic degradation [10]. This has previously been demonstrated to occur with transition metal oxides. For example, dextran coated iron oxide nanoparticles can be readily internalized by a number of different cell types *in vitro*, including lymphoblastoid B (TK6), lung epithelial (16HBE14o^−^), and macrophages (differentiated THP-1) cells, often localized within lysosomes [11, 12]. Subsequent analysis demonstrated that the material induced DNA damage (quantified by the *in vitro* micronucleus assay) via oxidative stress. This was initiated by metal ions promoting hydroxyl radical formation, consequently resulting in the formation of toxic DNA adducts capable of causing DNA strand breakage [13]. Conversely NM uptake into a cell does not always elicit a toxicological response, some carbon nanotubes for instance have been shown to undergo cellular uptake with no acute toxic response [4]. Moreover, toxicological assessment of an industrially relevant titanium dioxide (TiO_2_) (nano)particle panel, which demonstrated cellular uptake to be dependent on size and crystalline state, showed no toxicological response to any of the test materials regardless of uptake potential [14]. Most *in vitro* studies that have correlated cellular uptake with genotoxicity utilize traditional assays such as the *in vitro* micronucleus or hypoxanthine phosphoribosyl transferase (HPRT) assays [15]. There are however various international initiatives to make a paradigm shift in toxicity testing toward novel approaches that integrate mechanistic evaluation [16]. ToxTracker is a new approach method (NAM), mouse embryonic stem cell-based (mES) assay that identifies genotoxic compounds using six fluorescent reporter genes specifically activated by different cellular signaling responses. Responses are associated with both direct and indirect genotoxicity [17] and provide insight into the mechanisms involved, including oxidative damage, protein damage, and p53 activation. The assay has undergone validation [18] and has been used previously to assess the potential genotoxicity of nanoparticle metals and oxides [19–21].

The aim of this current study was to investigate the cellular uptake and toxicological endpoints using transmission electron microscopy (TEM) along with the *in vitro* ToxTracker assay to assess the toxic and genotoxic effects of 10 (nano)materials with differing physico-chemical properties. The NMs were: TiO_2_-NM102, TiO_2_-NM105, polystyrene (50 nm), Silica (50 nm), polyethylene (200—9900 nm), carbon black (20 nm), gold nanorods, tungsten carbide/cobalt, tungsten carbide, and TiO_2_ E171. These materials were selected to provide a broad range of industrially relevant samples from a range of different sectors, including food, medicine, and automotive.

## Materials and methods

All test materials are listed in Table 1.

**Table 1:** Test materials used, provider, product number, and further information provided by supplier including refractive index (n’).

**Material**	**Provider**	**Order number**	**Description (if provided by supplier)**
**TiO**_**2**_ **-NM102**	JRC nanomaterial repository (Italy)	JRCNM10202	16–40 nm, impurities (ICP-OES) S, Ca, Zr, K, Na, P, W. n’ 2.48
**TiO**_**2**_ **-NM105**	JRC nanomaterial repository (Italy)	JRCNM01005	10–45 nm, impurities (ICP-OES) Na. n’ 2.48
**TiO**_**2**_ **(E171)**	Sigma Aldrich (Germany)	1.00805	EMPROVE Essential, LOT: K54475005. n’ 2.48
**Polystyrene (50 nm)**	Polysciences (USA)	07307–15	Polybeads 36–64 nm, 2.5% solids. n’ 1.59
**Silica (50 ± 10 nm)**	Polysciences (USA)	24 040	Colloidal SIO_2,_ 50 nm. 10% solids. n’ 1.47
**Polyethylene (200-9900 nm)**	Cospheric LLC (USA)	PENS-0.95200-9900 nm—1 g	200–9900 nm, purity >70%. n’ 1.51
**Carbon Black (20 nm)**	Nanografi (Turkey)	NG04EO0709	20nmm, pH 2.4, surface area 195 m^2^/g. n’ 1.95
**Gold Nanorods (40 ± 15 nm)**	Clinisciences (Netherlands)	GRCH660-500 U	NanoXact Gold Nanorods—Bare (Citrate)—Peak @ 660 nm · 48 nm × 18 nm, 50 OD in water—500 μl. n’ 0.2
**Tungsten Carbide/Cobalt (<200 nm)**	NanoAmor (USA)	5561HW	Batch: 5561–072018. Purity: 99.5%. 8 wt% Co/WC <200 nm. n’ 2.13
**Tungsten Carbide (50 nm)**	NanoAmor (USA)	5551BD	Batch: 5551–042622. Purity: 99.9%. WC, 50 nM. n’ 2.42

Prior to all experiments, the test materials were prepared in cell culture medium (DMEM) without serum [cell experiments and dynamic light scattering (DLS)] or water (for DLS). For every sample, a 1 mg/ml stock was prepared, which was sonicated for 10 min twice in a Branson2800 sonicating water bath, model CPX2800H, 40 kHz, except for the gold nanorods, which were already in suspension. Fresh samples were prepared for every exposure and used within 30 min.

### Dynamic light scattering sizing of test materials

DLS was performed using a ZetaSizer Pro Blue (Malvern Instruments, UK). The test materials (Table 1) were diluted to a concentration of 100 μg/ml and incubated at 37°C for 1 h prior to undertaking measurements and were maintained at this temperature until used. Dilutions were carried out in both 10% serum-containing media (DMEM with 10% fetal bovine calf serum) and H_2_O; 1 ml of each dispersant was used in 1.5 ml Eppendorf tubes. The DLS standard operating procedure was set up according to the instrument’s operating manual. This required the input of dispersant dynamic viscosity (cP) (water: 0.6963, media: 0.7078) and the refractive index of the test materials (Table 1). Then, 100 μl of the samples were pipetted into micro cuvettes (Sigma-Aldrich, UK) and inserted into the instrument. The instrument was set to equilibrate the sample at 37°C for 2 min prior to measurement initiation and maintained this temperature throughout the measurement process. No visible precipitation was observed during the measurement process. Each measurement consisted of 10 runs, which were averaged, and each sample was performed in triplicate (individually prepared). The instrument attenuator was set to automatic, and analysis of the resulting data was conducted using ZS Xplorer version 3.22 (Malvern, UK). The data were presented as a *z*-average (nm), a size range (nm), and a polydispersity index (PDI).

### ToxTracker analysis

The protocol for performing the ToxTracker assay has been published previously [17,22]. Briefly, the six independent mES reporter cell lines were seeded in gelatin-coated 96-well cell culture plates in 200 μl mES cell medium (50 x 10^3^ cells per well) and incubated at 37°C, 5% CO_2_ for 24 h. Following the cell seeding into the 96-well plates, medium was aspirated and fresh mES cell medium containing 10% fetal calf serum (FCS) and the diluted materials were added to the cells. For the tested materials, five concentrations were tested in 2-fold dilutions. Test concentrations were selected based on cytotoxicity data from a concentration range finding experiment (data not included). Three independent repeat experiments were performed, and in each experiment, one well per concentration was measured for every cell line. In each well, at least 5000 intact cells were measured. Induction of the green fluorescent protein (GFP) reporters and change in side scatter was determined after 24 h exposure using a flow cytometer (Guava EasyCyte—Merck Millipore). Cells were collected by trypsinization after washing twice in phosphate buffered saline (PBS) and resuspended in PBS+ 2% FCS. Only GFP expression and mean side scatter in intact single cells was determined. Mean GFP fluorescence was measured and used to calculate GFP reporter induction compared to a vehicle control treatment. Cytotoxicity was estimated by cell count after 24 h exposure using a flow cytometer and was expressed as fraction of intact cells (based on forward and side scatter) after 24 h exposure compared to vehicle exposed controls. For cytotoxicity assessment in the ToxTracker assay, the relative cell survival for the six different reporter cell lines was averaged because the cytotoxicity levels were very similar. Positive reference treatments with cisplatin (DNA damage), diethyl maleate (oxidative stress), and tunicamycin (unfolded protein response) were included in all experiments. Data were classified according to the criteria defined in the interlaboratory validation [18].

### Cell preparation and resin embedding for transmission electron microscopy

0.8 million parental mES cells were seeded in 6-well plates in a single replicate. 24 h after seeding, cells were exposed for 24 h to the NP at a concentration of 25 μg/ml. After 24 h exposure, cells were harvested using trypsinization, pelleted (230 g for 10 min), and washed once in maintenance buffer (0.1 M sucrose, 200 mM di-sodium hydrogen orthophosphate dihydrate, and 200 mM sodium dihydrogen orthophosphate monohydrate in double distilled H_2_O). Cells were then post-fixed in 1% osmium tetroxide fixative (2.26% sodium dihydrogen orthophosphate, 2.52% sodium hydroxide, 5.4% glucose, and 1% osmium tetroxide) for 1.5 h at 4°C in the dark on a rocker (30 rpm). After secondary fixation, cells were re-pelleted at 230 g for 10 min and the fixative aspirated off. At this stage, the TAAB Premix resin kit (TAAB Laboratory and Microscopy Reagents, UK) was prepared by the addition of the hardener to the resin and placed on a roller for 1 h and the accelerator component was added. Prior to adding resin to cell pellets, dehydration stages were undertaken whereby cells were placed in 10% ethanol for 10 min, 70% ethanol for 30 min, and then twice in 100% ethanol for 20 min; all dehydration stages were undertaken under gentle agitation. Cell samples were subsequently placed into 100% propylene oxide for 20 min (twice), then placed in 1:1 ratio of resin and propylene oxide for 90 min, finally cells were placed into 100% resin overnight at 4°C. Resin was pre-warmed at room temperature on a roller for 1 h, and then the cell sample resin was replaced with 100% fresh resin). Cell samples were placed in an oven at 60°C for 24 h with the caps left open (to allow any residual propylene oxide to evaporate).

### Sectioning by ultramicrotomy

The resin blocks were cut free from the 0.5 ml Eppendorf tube and trimmed with glass knives using an EM-UC7 ultramicrotome (Leica Microsystems, UK) at 100 mm/s approach distance set at 150 nm until the blocks had a flat face edge that was encompassing the cell pellet. From this block face, a raised mesa was cut with the dimensions 750 μm x 750 μm and 50 μm deep at 100 mm/s and approach set at 100 nm. Sections, 70 nm thick, were then cut from this mesa using an Ultra 45° diamond knife (Diatome, Switzerland). The cutting speed was set at 1 mm/s, and the approach distance was set at 70 nm. Sections were floated out onto a water bath (part of the diamond knife component) and picked up onto carbon-coated 150 mesh copper grids (Agar Scientific, UK) held in 0.07 mm tipped self-closing tweezers (Agar Scientific, UK).

### Transmission electron microscopy imaging

Samples were examined using a FEI TALOS (ThermoFisher, UK) equipped with a field emission gun operated at 200 kV accelerating voltage. An Oxford Instruments INCA 350 EDX system/80 mm X-Max SDD detector was used to measure the energy-dispersive X-ray (EDX) spectra and the images were captured on a Gatan Orius SC600A CCD camera. Where the presence of the test material was suspected within the tissue sections, elemental analysis was undertaken using EDX to provide an elemental spectrum of the observed object. For each sample 100 cells were imaged from five different areas selected at random, to avoid scoring bias.

### Data and statistical analysis

All data are presented as the mean ± SD, except for ToxTracker data, where data are represented as mean ± SEM. All analysis was completed over three independent biological replicates (*n* = 3). To assess differences in mean side scatter, a one-way ANOVA with Dunnett’s test for multiple comparisons was used in GraphPad Prism version 10 (Dotmatics). Here, a *P* < .001 was considered significant.

## Results

This investigation aimed to assess the potential genotoxicity of 10 (nano)materials; titanium Dioxide (TiO_2_)-NM102, TiO_2_-NM105, polystyrene (50 nm), silica (50 nm), polyethylene (200−9900 nm), carbon black (20 nm), gold nanorods, tungsten carbide/cobalt, tungsten carbide, and TiO_2_ (E171) in the ToxTracker assay. Additionally, the ability of these materials to undergo cellular uptake in the mES reporter cell line was assessed by electron microscopy.

### Characterization of test materials agglomeration in water and cell culture medium

Size measurements of the test materials were undertaken by DLS in water and complete cell culture medium to evaluate the agglomeration of the test material under the experimental conditions (Table 2). In general, a PDI of <0.7 is deemed acceptable to provide an accurate size measurement of a test sample. All test materials except tungsten carbide–cobalt in complete cell culture medium met this requirement.

**Table 2:** DLS sizing measurements of test materials in water and cell culture medium, the measurement has been displayed as an average (*Z*-average) (nm), a size range (nm), and a PDI (*n* = 3) (± = standard deviation of the mean).

**Material**	**Z-average (nm) water**	**Z-Average (nm) media**	**Size range (nm) water**	**Size range (nm) media**	**PDI water**	**PDI media**
TiO_2_– NM102	1014 ± 198.6	1614.0 ± 469.7	828.70–1402.0	763–2170	0.14 ± 0.056	0.459 ± 0.17
TiO_2_– NM103	466.4o ± 50.75	534.80 ± 40.93	376.20–534.50	459.4–619.4	0.34 ± 0.060	0.490 ± 0.05
Polystyrene (50 nm)	39.18 ± 1.29	37.99 ± 3.456	22.68–23.41	28.17–41.6	0.515 ± 0.01	0.507 ± 0.02
Silica (50 ± 10 nm)	22.93 ± 0.26	22.90 ± 0.298	22.68–23.41	22.57–23.54	0.547 ± 0.04	0.535 ± 0.01
Polyethylene (200—9900 nm)	72.24 ± 168.80	729.10 ± 604.10	13.7–522.3	400.69–2066	0.354 ± 0.10	0.457 ± 0.26
Carbon Black (20 nm)	997.60 ± 366.10	555.30 ± 470.0	600.2–1674	87.92–1441	0.664 ± 0.14	0.59 ± 0.12
Gold Nanorods (40 ± 15 nm)	16.07 ± 2.36	14.38 ± 0.98	14.41–22.12	14.09–17.05	0.385 ± 0.08	0.40 ± 0.03
Tungesten Carbide/Cobalt (<200 nm)	483.70 ± 191.20	521.10 ± 242.5	260–818.6	46.54–1242.2	0.754 ± 0.11	0.59 ± 0.07
Tungesten Carbide (50 nm)	324.1 ± 15.84	217.0 ± 9.37	307.7–356.8	202.6–229.5	0.210 ± 0.01	0.32 ± 0.03
TiO_2_ E171	1406.0 ± 659.20	1658.0 ± 688.3	694.8–2715	621–2582	0.1595 ± 0.59	0.64 ± 0.14

TiO_2_-NM102, TiO_2_ NM-103, TiO_2_ E171, polyethylene, and tungsten carbide cobalt all demonstrated an increase in agglomerate size in culture media compared to water. Conversely, carbon and tungsten carbide demonstrated a decrease in agglomerate size in culture media. Polystyrene, gold nanorods, and silica maintained a narrow size distribution in both dispersants with limited or minor variation in agglomerate sizing (between culture media and water).

### Assessment of potential genotoxicity and cellular stress response upon nanomaterial exposure

Toxicological evaluation was undertaken in mES cells following treatment with the 10 selected NMs suspended in cell culture medium. Freshly prepared suspensions were sonicated to disperse the NMs in serum-free medium to avoid foam, and serum was added before exposure. After 24 h of exposure, cells were harvested, and cell numbers, GFP signal (reporter induction), and mean side scatter were measured using flow cytometry.

**Figure 1:**
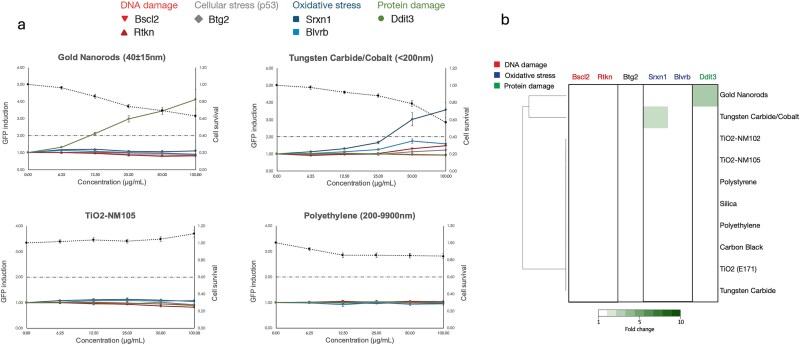
(a) ToxTracker analysis of gold nanorods, tungsten carbide/cobalt, TiO_2_-NM105, and polyethylene showing change in cytotoxicity, DNA damage, cellular stress (p53), oxidative stress, and protein damage reporters in mES cell line, and (b) heatmap showing ToxTracker response for all tested NMs with hierarchical clustering based on response.

Regardless of test material, exposure of mES cells to the NMs did not result in activation of the ToxTracker DNA damage reporters Bscl2-GFP and Rtkn-GFP. Activation of the oxidative stress reporter Srxn1-GFP was observed upon exposure to tungsten carbide cobalt, but exposure to this material did not activate the second reporter for oxidative stress, Blvrb-GFP, more than 2-fold (Fig. 1a). Activation of the Ddit3-GFP protein stress reporter was observed only after exposure to the gold nanorods. Exposure to the other test substances did not result in activation of any of the ToxTracker reporters (Fig. 1b). Limited cytotoxicity was observed in mES cells treated with polyethylene (15%) and gold nanorods (35%) (100 μg/ml treatment only). Moderate cytotoxicity was observed when treated with tungsten carbide/cobalt (45%) at the 100 μg/ml dose.

**Figure 2:**
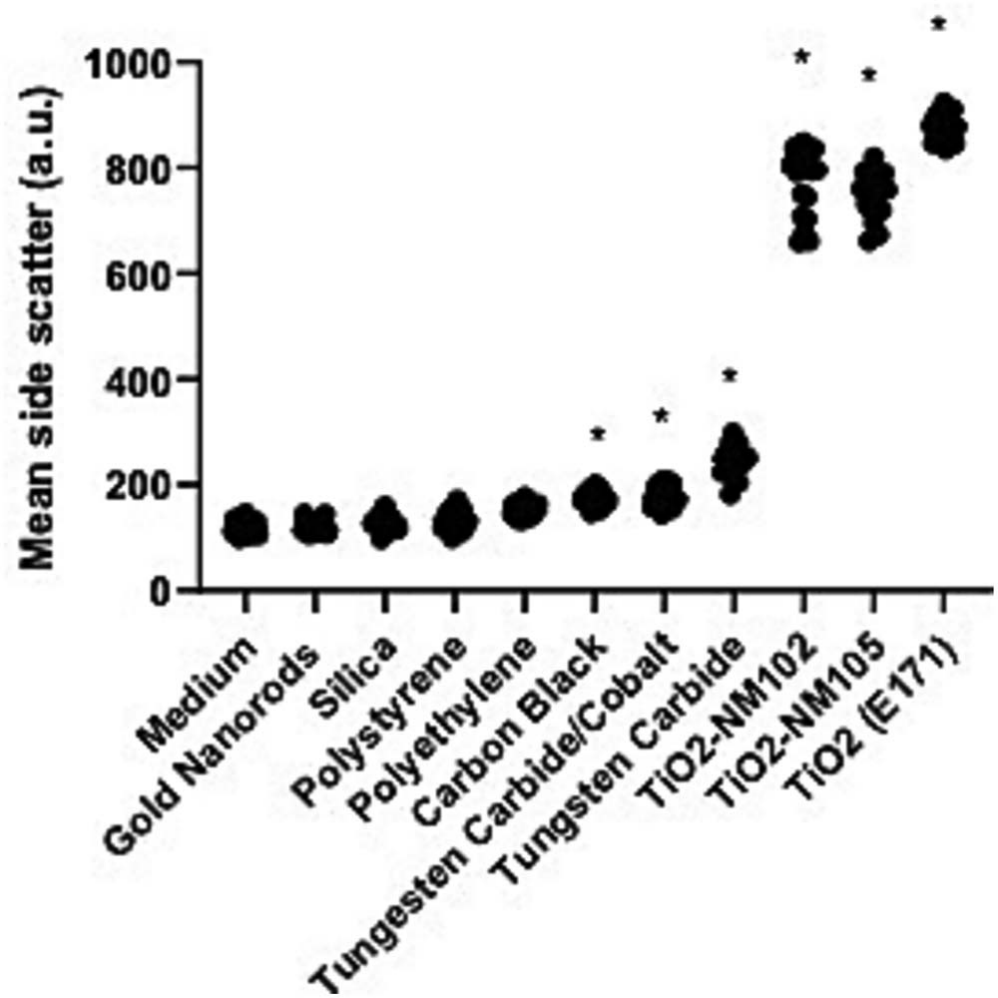
Quantification side scatter. Individual values (*n* = 18 wells, at least 5000 cells/well) of mean side scatter at 100 μg/ml shown. *P < .001 in one-way ANOVA with Dunnett’s test for multiple comparisons.

Side scatter of cells measured with flow cytometry increases with increasing cell complexity and can indicate interaction of the cells with nanoparticles. However, interaction does not necessarily result in cellular uptake. Increases in side scatter observed at the highest tested concentration (100 μg/ml) were determined compared to background (medium exposure only) (Fig. 2). Exposure to the three TiO_2_ samples resulted in the highest increase in side scatter. A clear increase in side scatter was also observed for carbon black, tungsten carbide, and tungsten carbide/cobalt. This increase in side scatter suggests surface association or internalization, since it was not washed away during harvest, but it is not possible to distinguish between internalized material and material on the outside of the cell.

### Evaluation of nanomaterial internalization by mES following exposure

Subcellular uptake analysis was undertaken in mES cells following treatment with the NMs by TEM. Cellular analysis of mES cells treated with TiO_2_-NM102 polyethylene, gold nanorods, tungsten carbide, and tungsten carbide cobalt, and TiO_2_ (E171) by TEM did not show any visible evidence of cellular uptake. A representative image of an mES cell that has not taken up any test material is shown in Supplementary Fig. S1.

**Figure 3:**
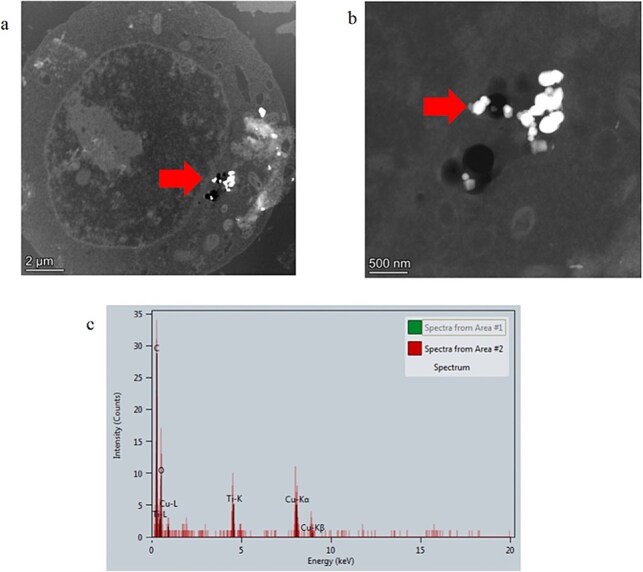
TEM cellular uptake assessment of test material TiO_2_ NM105 in parental mES cells. (a) Whole mES cell image with evidence of internalized material, (b) higher magnification TEM image of material in mES cell, and (c) EDX spectra of material confirming presence of titanium. Arrows identify location of test material.

In contrast, ~25 mES cells treated with TiO_2_-NM105 were imaged from a total of five areas on one TEM grid. When all cell sections were imaged, there was evidence of the test material localized within the cytoplasm of ~60% of cells imaged (Fig. 3a and b). The material appeared to be highly electron-dense, crystalline, and faceted; EDX analysis confirmed the material to consist of titanium (Fig. 3c).

**Figure 4:**
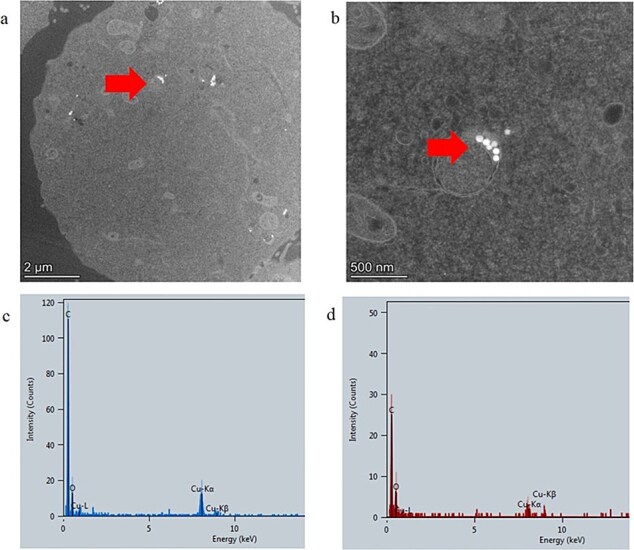
TEM cellular uptake assessment of test material polystyrene in mES cells. (a) Whole mES cell image with evidence of internalized material, (b) higher magnification TEM image of material in a mES cell localized in a membrane-bound vesicle, (c) EDX analysis of material showing increased carbon composition compared to cellular background shown in (d). Arrows identify the test material.

**Figure 5:**
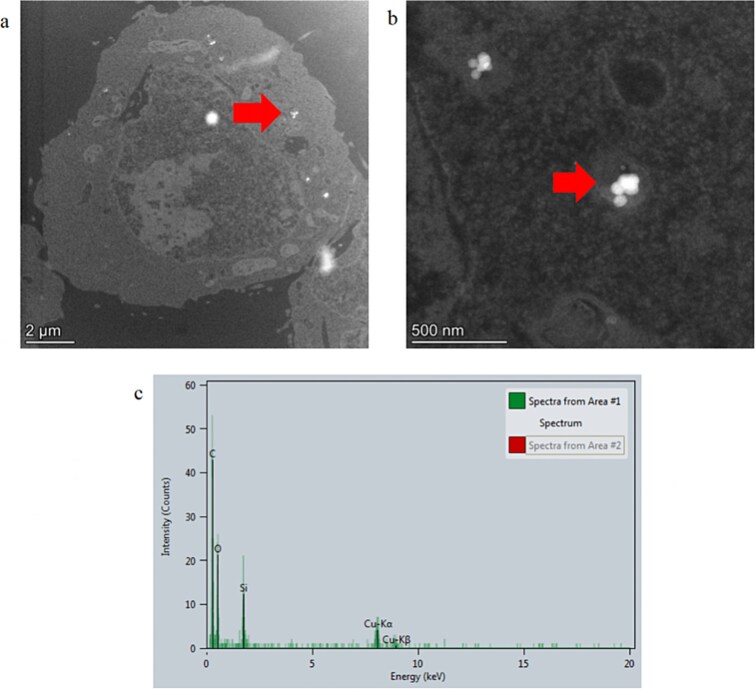
TEM cellular uptake assessment of silica in mES cells. (a) Whole mES cell image with evidence of internalized material, (b) higher magnification TEM image of material in a mES cell, and (c) EDX spectra of material confirming presence of silica. Arrows identify location of test material.

TEM analysis of mES cells treated with polystyrene nanoparticles showed evidence of the material in ~70% of imaged cells (confirmed by EDX). The particles were localized within membrane-bound vesicles or free within the cytoplasm (Fig. 4a and b). Silica was also shown to be present in 70% of cells imaged, localized in the cytoplasm (Fig. 5a and b). This was confirmed by EDX analysis demonstrating the presence of silica where the internalized material was observed (Fig. 5c).

**Figure 6:**
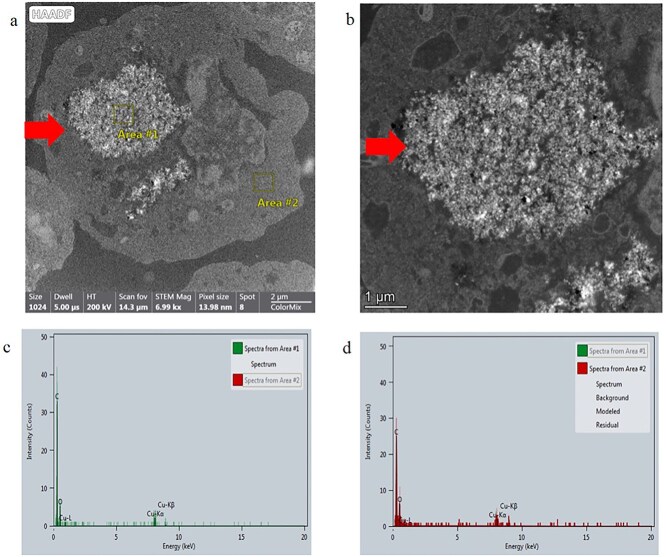
TEM cellular uptake assessment of carbon black in mES cells. (a) Whole mES cell image with evidence of internalized material, (b) higher magnification TEM image of material in a mES cell, (c) EDX spectra of material confirming greater intensity of carbon in Area 1 compared to Area 2 (in sub figure a), and (d) EDX spectra of Area 2 in sub figure a.

During TEM analysis of mES cells treated with carbon black, electron-dense particulate matter was observed within 10% of the cells imaged (Fig. 6a and b). The material appeared to be electron dense and with an irregular shape. EDX analysis was performed across two regions of the cell where uptake was suspected and an area of cytoplasm where no material was visible (areas 1 and 2 in Fig. 6a, respectively) (EDX shown in Fig. 6c and d). This analysis showed the electron-dense region to have a higher carbon intensity than the normal cell cytoplasm, therefore confirming the material to be comprised of carbon.

## Discussion

This study undertook (geno)toxicological assessment of a panel of NMs by application of the ToxTracker assay. This was correlated with subcellular uptake evaluation by electron microscopy, providing a visual assessment of the test material’s capacity to enter the test cell line, and its intracellular fate. Considering this, the study herein assessed the cellular response to the test materials using the ToxTracker assay, focusing on genotoxicity, oxidative stress, and cell death [23]. None of the tested materials activated the ToxTracker DNA damage reporters, and therefore all would be classified as non-genotoxic in the assay. For only two materials, activation of any of the other ToxTracker reporters was observed: tungsten carbide cobalt activated the oxidative stress response, and gold nanorods activated the protein stress response. However, it is important to consider these responses together with the relevant cellular uptake data.

Genotoxicity evaluation was complemented with an assessment of the ability of the materials to undergo cellular uptake in the mES cell line by electron microscopy analysis. TEM analysis is considered the gold standard in confirming the interaction and internalization of test material within mammalian cells. This approach allows for ultrastructural identification of NM internalization within single cells [24]. NMs, by their nature, have significant potential to be internalized by target cells both *in vitro* and *in vivo* dependent on their physico-chemical properties. Information on the ability of a test NM to undergo cellular uptake and its localization within the cell is key in understanding its toxicological fate. Cellular uptake did not result in a genotoxic response, as 4/10 test materials were visibly internalized by the test cell line, yet none of them were genotoxic in the test cell line. On the other hand, ToxTracker indicated biological effects of some NMs that were not internalized. This would possibly be indicative of extracellular effects such as cell surface interactions and/or ion dissolution within the cell culture medium (i.e. biological interactions not detectable by TEM) [14]. Tungsten carbide cobalt, for instance, caused moderate cytotoxicity but was not internalized by the test cell line. The cells treated with this material did, however, demonstrate increased side scatter during ToxTracker analysis, indicative of cell surface association.

TiO_2_-MN105 did not promote any response to the ToxTracker assay despite being visible in the cytoplasm in 60% of cells imaged by TEM. TiO_2_ cellular uptake *in vitro* has been shown to be highly cell type-specific, with a previous study showing that lung epithelial cells (A549) were able to internalize multiple different TiO_2_ types of both nano and micron size [14]. Whereas the suspension lymphoblast cell line TK6 only internalized TiO_2_ in the anatase form with a primary particle size <160 nm. A study by Allouni investigating how TiO_2_ crystalline state impacts cellular uptake potential (in fibroblasts), found that anatase TiO_2_ has a greater affinity for the cell surface and consequently has greater uptake potential [25]. The likelihood of cell membrane association was thought to be related to differing surface energies in anatase’s distorted tetragonal atomic arrangement [26]. TiO_2_-NM105, comprised of both anatase and rutile forms, can conform to this potential theory [27]. However, the second TiO_2_ tested in this current study, TiO_2_-NM102, was pure anatase, and no uptake of this particle type was observed in mES cells. This difference in uptake potential may be attributed to their differing average hydrodynamic diameters under experimental conditions, with TiO_2_-NM102 forming significantly larger agglomerates (1614 nm) than TiO_2_-NM105 (534.8 nm). The larger agglomerate size of TiO_2_-NM102 presumably limited or inhibited the potential of the material to enter the cells via Clathrin- and caveolin-mediated mechanisms, as highlighted by Rennick *et al*. [28], these pathways usually account for particle (diameter) sizes ≤200 nm. For both materials, a substantial increase in side scatter was observed, but neither of the test TiO_2_ variants tested caused any significant toxic response, with only TiO_2_-NM102 causing a very low level of cytotoxicity at the highest applied dose (100 μg/ml). The increases in side scatter by TiO_2_-NM102 and TiO_2_-NM105, despite lack of evidence of cell uptake (by TEM), is consistent with a previous study showing that some forms of TiO_2_ interact at the cell surface [14]. The ability of TiO_2_ to cause cytotoxicity and genotoxicity is varied across the literature and dependent on the test material physico-chemical properties, exposure system used, and endpoint assays applied [29–31].

### Effects of nanomaterial size and shape

*In situ* material physico-chemical characterization is important for the genotoxicity assessment of NMs. Consequently, this study undertook DLS sizing analysis of all test materials. Except for polystyrene, gold nanorods, and silica, all the test materials deviated significantly in size when dispersed in culture media compared to supplier-provided information on the primary particle size. This is an expected phenomenon due to mutual attraction of individual units of material by van der Waals forces causing agglomeration in solution (dependent on material surface chemistry) [32]. All test materials fell within a PDI range for the measurement to be considered acceptable (<0.7); this indicated that DLS was an appropriate technique for the sizing evaluation undertaken [33]. NM agglomeration in an *in vitro* exposure system will greatly influence its effect at the cellular level, affecting interactions at the cell surface, uptake potential, and intercellular interactions [34]. It was interesting to compare the DLS measurements of all three TiO_2_ types and indeed all the other test materials to polystyrene nanoparticles under experimental conditions. Polystyrene is an aromatic hydrocarbon and a versatile plastic used in many consumer products [35]. Polystyrene nanoparticles are often used as a model material for uptake studies due to their consistent size, shape, surface charge and limited potential to agglomerate in solution, which can easily facilitate entry into a target cell [36]. Indeed, sizing of this material in cell culture media showed limited to no agglomeration (size range: 28.17–41.6 nm), and only a limited cytotoxic response was noted in mES cells treated with the highest dose of the material. However, cellular uptake was observed in 70% of the cells imaged, showing a high internalization potential. Polystyrene uptake can be accounted for firstly by particle size; the DLS measurements put the test material in a size range for uptake by endocytic mechanisms [37]. Moreover, the TEM images of the particles within mES cells, show them to be nearly spherical. In essence the more attuned a test material is to a sphere, the greater the likelihood of it being taken up into a cell. This is because cellular uptake is dependent on four major factors: size, internalization force, membrane barrier, and the chemical barrier [38]. If a material is more spherical it is more likely to overcome the physical barrier and internalize at a faster rate, as a sphere requires less membrane binding energy than a material with a high aspect ratio [39]. This fact is also consistent with the degree of uptake of silica into mES cells, observed to be 70% of imaged cells. Although not as spherical as polystyrene, its observed shape likely facilitates its cellular uptake. No toxicity was noted in cells treated with silica, but its degree of uptake was consistent with previous studies [39]. Material shape would also play a significant factor in the lack of uptake observed in mES cells treated with gold nanorods, tungsten carbide, polyethylene, and tungsten carbide/cobalt. The gold nanorods, despite falling within the sizing range (14.09–17.05 nm) for active cellular uptake, were likely prevented from entering cells due to their high aspect ratio [39, 40].

### Toxic responses

TEM imaging of mES cells treated with carbon black appeared to demonstrate visible cellular uptake, despite no observed effect when evaluated by the ToxTracker system. Many studies have demonstrated that carbon black causes oxidative stress, genotoxicity, and chronic immune responses [41–43]. These toxic responses are often exacerbated by or attributed to surface-bound metals and polycyclic hydrocarbons, potentially accounting for variability in the toxic outcome of carbon black exposure [44]. Elemental analysis (EDX) of the internalized carbon black in this study did not demonstrate the presence of any metal contaminants. Similarly to carbon black, the toxicity of polyethylene is of concern from an environmental exposure perspective. The test polyethylene used here did not show any toxic response and none of the material was shown to undergo cell internalization. Historically, polyethylene is classified as inert, chemically neutral, and of low toxicological risk; however, risk from this substance can occur from chronic exposure and following combustion [45–47].

Consistent with earlier results, the ToxTacker assay did not indicate that any of the test materials caused genotoxicity, and most materials caused no or a very limited cytotoxic response. The only exception to this was tungsten carbide/cobalt which elicited moderate cytotoxicity (45%) at the highest applied dose (100 μg/ml). No cellular uptake of this material was observed in mES cells and, consequently, cannot be attributed to its cytotoxic potential. However, the ToxTracker system also indicated that the material promoted an oxidative stress response. Cobalt is a transition metal; if its ions are released into a biological system there is the potential for the formation of oxidative radicals by Fenton reactions leading to downstream oxidative stress [48]. Ion release into the cell would not be detectable by electron microscopy. Exposure to soluble cobalt materials has been shown to activate the oxidative stress response in ToxTracker [49]. It should also be noted that the genotoxicity of tungsten-carbide cobalt *in vitro* appears to be cell line dependent. When tested on the TK6 (human lymphoblast b cell line) and primary human lymphoblast, tungsten carbide cobalt promotes a significant degree of chromosomal damage when quantified by the *in vitro* micronucleus assay [50].

## Conclusion

This study demonstrated that TiO_2_-NM105, silica, polystyrene, carbon black, and tungsten carbide are non-genotoxic to the mES cell line *in vitro* despite undergoing cell uptake in the ToxTracker cells. A continued focus is needed to supplement NM genotoxicity studies with cellular uptake analysis. This study highlighted the importance of such evaluations using TEM for uptake assessment. Whilst analysis such as side scatter from flow cytometry data can provide useful cellular interaction information, it is unable to confirm material internalization. Materials on the nanoscale present different challenges when evaluating their toxic potential; uptake analysis can help provide a key insight into understanding fate and interpreting the outcomes of hazard characterization endpoints.

## Supplementary Material

Supplementary_Figs_geaf013

## Data Availability

The data generated in this manuscript are available from the corresponding author upon reasonable request.

## References

[1] Doak SH, Andreoli C, Burgum MJ et al. Current status and future challenges of genotoxicity OECD Test Guidelines for nanomaterials: a workshop report. *Mutagenesis* 2023;38:183–91. 10.1093/mutage/gead017 37234002 PMC10448853

[2] Evans SJ, Vecchiarelli PM, Clift MJD et al. Overview of nanotoxicology in humans and the environment; developments, challenges and impacts. In: Lead JR, Doak SH, Clift MJD (eds.), *Nanotoxicology in Humans and the Environment*, pp. 1–40. Cham: Springer International Publishing, 2021.

[3] Unfried K, Albrecht C, Klotz L-O et al. Cellular responses to nanoparticles: target structures and mechanisms. *Nanotoxicology* 2007;1:52–71. 10.1080/00222930701314932

[4] Augustine R, Hasan A, Primavera R et al. Cellular uptake and retention of nanoparticles: insights on particle properties and interaction with cellular components. *Mater Today Commun* 2020;25:101692. 10.1016/j.mtcomm.2020.101692

[5] Elespuru R, Pfuhler S, Aardema MJ et al. Genotoxicity assessment of nanomaterials: recommendations on best practices, assays, and methods. *Toxicol Sci* 2018;164:391–416. 10.1093/toxsci/kfy100 29701824

[6] Rolo D, Tavares A, Vital N et al. Overview of adverse outcome pathways and current applications on nanomaterials. In: Louro H, Silva MJ (eds.), *Nanotoxicology in Safety Assessment of Nanomaterials*, pp. 415–39. Cham: Springer International Publishing, 2022. 10.1007/978-3-030-88071-2_17 35583654

[7] Wu K, Zhou Q, Ouyang S. Direct and indirect genotoxicity of graphene family nanomaterials on DNA—a review. *Nanomaterials* 2021;11:2889. 10.3390/nano11112889 34835652 PMC8625643

[8] Horie M, Tabei Y. Role of oxidative stress in nanoparticles toxicity. *Free Radic Res* 2021;55:331–42. 10.1080/10715762.2020.1859108 33336617

[9] MacCormack TJ, Clark RJ, Dang MK et al. Inhibition of enzyme activity by nanomaterials: potential mechanisms and implications for nanotoxicity testing. *Nanotoxicology* 2012;6:514–25. 10.3109/17435390.2011.587904 21639725

[10] Rathore B, Sunwoo K, Jangili P et al. Nanomaterial designing strategies related to cell lysosome and their biomedical applications: a review. *Biomaterials* 2019;211:25–47. 10.1016/j.biomaterials.2019.05.002 31078050

[11] Singh N, Jenkins GJS, Nelson BC et al. The role of iron redox state in the genotoxicity of ultrafine superparamagnetic iron oxide nanoparticles. *Biomaterials* 2012;33:163–70. 10.1016/j.biomaterials.2011.09.087 22027595

[12] Evans SJ, Clift MJ, Singh N et al. In vitro detection of secondary mechanisms of genotoxicity induced by engineered nanomaterials. *Part Fibre Toxicol* 2019;16:1–14. 10.1186/s12989-019-0291-7 30760282 PMC6374901

[13] Carter A, Racey S, Veuger S. The role of iron in dna and genomic instability in cancer, a target for iron chelators that can induce ros. Applied Sciences 2022;12:10161. 10.3390/app121910161

[14] Evans SJ, Lawrence RL, Ilett M et al. Industrial-relevant TiO_2_ types do not promote cytotoxicity in the A549 or TK6 cell lines regardless of cell specific interaction. *Toxicol in Vitro* 2022;83:105415. 10.1016/j.tiv.2022.105415 35752104

[15] Liu BM, Hayes AW. Mechanisms and assessment of genotoxicity of metallic engineered nanomaterials in the human environment. *Biomedicines* 2024;12:2401. 10.3390/biomedicines12102401 39457713 PMC11504605

[16] Groenewold M, Bleeker EAJ, Noorlander CW et al. Governance of advanced materials: shaping a safe and sustainable future. *NanoImpact* 2024;35:100513. 10.1016/j.impact.2024.100513 38821170

[17] Hendriks G, Derr RS, Misovic B et al. The extended ToxTracker assay discriminates between induction of DNA damage, oxidative stress, and protein misfolding. *Toxicol Sci* 2016;150:190–203. 10.1093/toxsci/kfv323 26719371 PMC5009621

[18] Hendriks G, Adriaens E, Allemang A et al. Interlaboratory validation of the ToxTracker assay: an in vitro reporter assay for mechanistic genotoxicity assessment. *Environ Mol Mutagen* 2024;65:4–24. 10.1002/em.22592 38545858

[19] Zhao JH, Stacey D, Eriksson N et al. Genetics of circulating inflammatory proteins identifies drivers of immune-mediated disease risk and therapeutic targets. *Nat Immunol* 2023;24:1540–51. 10.1038/s41590-023-01588-w 37563310 PMC10457199

[20] Yaeger R, Cowell E, Chou JF et al. RAS mutations affect pattern of metastatic spread and increase propensity for brain metastasis in colorectal cancer. *Cancer* 2015;121:1195–203. 10.1002/cncr.29196 25491172 PMC4523078

[21] Cappellini F, Hedberg Y, McCarrick S et al. Mechanistic insight into reactivity and (geno)toxicity of well-characterized nanoparticles of cobalt metal and oxides. *Nanotoxicology* 2018;12:602–20. 10.1080/17435390.2018.1470694 29790399

[22] Thakkar Y, Moustakas H, Moelijker N et al. Utility of ToxTracker in animal alternative testing strategy for fragrance materials. *Environ Mol Mutagen* 2023;64:234–43. 10.1002/em.22532 36762970

[23] Hendriks G, Atallah M, Morolli B et al. The ToxTracker assay: novel GFP reporter systems that provide mechanistic insight into the genotoxic properties of chemicals. *Toxicol Sci* 2012;125:285–98. 10.1093/toxsci/kfr281 22003191

[24] Hondow N, Harrington J, Brydson R et al. STEM mode in the SEM: a practical tool for nanotoxicology. *Nanotoxicology* 2011;5:215–27. 10.3109/17435390.2010.535622 21090920

[25] Allouni ZE, Høl PJ, Cauqui MA et al. Role of physicochemical characteristics in the uptake of TiO_2_ nanoparticles by fibroblasts. *Toxicol in Vitro* 2012;26:469–79. 10.1016/j.tiv.2012.01.019 22300586

[26] Selloni A . Anatase shows its reactive side. *Nat Mater* 2008;7:613–5. 10.1038/nmat2241 18654584

[27] Rasmussen K, Mast J, De Temmermann P et al. Titanium dioxide, NM-100, NM-101, NM-102, NM-103, NM-104, NM-105: characterisation and physicochemical properties. 2014.

[28] Rennick JJ, Johnston AP, Parton RG. Key principles and methods for studying the endocytosis of biological and nanoparticle therapeutics. *Nat Nanotechnol* 2021;16:266–76. 10.1038/s41565-021-00858-8 33712737

[29] Medina-Cabrera EV, Rühmann B, Schmid J et al. Characterization and comparison of *Porphyridium sordidum* and *Porphyridium purpureum* concerning growth characteristics and polysaccharide production. *Algal Res* 2020;49:101931. 10.1016/j.algal.2020.101931

[30] Biola-Clier M, Beal D, Caillat S et al. Comparison of the DNA damage response in BEAS-2B and A549 cells exposed to titanium dioxide nanoparticles. *Mutagenesis* 2016;32:161–72. 10.1093/mutage/gew055 27803034

[31] Freire K, Ordóñez Ramos F, Soria DB et al. Cytotoxicity and DNA damage evaluation of TiO_2_ and ZnO nanoparticles. Uptake in lung cells in culture. *Toxicol Res* 2021;10:192–202. 10.1093/toxres/tfaa112 PMC8045598 33884170

[32] Zare Y . Study of nanoparticles aggregation/agglomeration in polymer particulate nanocomposites by mechanical properties. *Compos A: Appl Sci Manuf* 2016;84:158–64. 10.1016/j.compositesa.2016.01.020

[33] Rodriguez-Loya J, Lerma M, Gardea-Torresdey JL. Dynamic light scattering and its application to control nanoparticle aggregation in colloidal systems: a review. *Micromachines (Basel)* 2024;15:24. 10.3390/mi15010024 PMC10819909 38258143

[34] Hondow N, Brydson R, Wang P et al. Quantitative characterization of nanoparticle agglomeration within biological media. *J Nanopart Res* 2012;14:1–15. 10.1007/s11051-012-0977-322448125

[35] Loos C, Syrovets T, Musyanovych A et al. Functionalized polystyrene nanoparticles as a platform for studying bio–nano interactions. *Beilstein J Nanotechnol* 2014;5:2403–12. 10.3762/bjnano.5.250 25671136 PMC4311717

[36] Ekkapongpisit M, Giovia A, Follo C et al. Biocompatibility, endocytosis, and intracellular trafficking of mesoporous silica and polystyrene nanoparticles in ovarian cancer cells: effects of size and surface charge groups. *Int J Nanomedicine* 2012;7:4147–58. 22904626 10.2147/IJN.S33803 PMC3418080

[37] Foroozandeh P, Aziz AA. Insight into cellular uptake and intracellular trafficking of nanoparticles. *Nanoscale Res Lett* 2018;13:339. 10.1186/s11671-018-2728-6 30361809 PMC6202307

[38] Lin J, Miao L, Zhong G et al. Understanding the synergistic effect of physicochemical properties of nanoparticles and their cellular entry pathways. *Commun Biol* 2020;3:205. 10.1038/s42003-020-0917-1 32355216 PMC7192949

[39] Li Y, Kröger M, Liu WK. Shape effect in cellular uptake of PEGylated nanoparticles: comparison between sphere, rod, cube and disk. *Nanoscale* 2015;7:16631–46. 10.1039/C5NR02970H 26204104

[40] White BE, White MK, Nima Alsudani ZA et al. Cellular uptake of gold nanorods in breast cancer cell lines. *Nanomaterials (Basel)* 2022;12:937. 10.3390/nano12060937 PMC8953423 35335749

[41] Hakkarainen H, Salo L, Mikkonen S et al. Black carbon toxicity dependence on particle coating: measurements with a novel cell exposure method. *Sci Total Environ* 2022;838:156543. 10.1016/j.scitotenv.2022.156543 35679919

[42] Di Ianni E, Jacobsen NR, Vogel UB et al. Systematic review on primary and secondary genotoxicity of carbon black nanoparticles in mammalian cells and animals. *Mutat Res/Rev Mutat Res* 2022;790:108441. 10.1016/j.mrrev.2022.108441 36007825

[43] Ryu Y, Roh S, Joung YS. Assessing the cytotoxicity of aerosolized carbon black and benzo[a]pyrene with controlled physical and chemical properties on human lung epithelial cells. *Sci Rep* 2023;13:9358. 10.1038/s41598-023-35586-7 37291179 PMC10250308

[44] Lindner K, Ströbele M, Schlick S et al. Biological effects of carbon black nanoparticles are changed by surface coating with polycyclic aromatic hydrocarbons. *Part Fibre Toxicol* 2017;14:8. 10.1186/s12989-017-0189-1 28327162 PMC5361723

[45] Herold DA, Rodeheaver GT, Bellamy WT et al. Toxicity of topical polyethylene glycol. *Toxicol Appl Pharmacol* 1982;65:329–35. 10.1016/0041-008X(82)90016-3 7179288

[46] Yao Z, Seong HJ, Jang Y-S. Environmental toxicity and decomposition of polyethylene. *Ecotoxicol Environ Saf* 2022;242:113933. 10.1016/j.ecoenv.2022.113933 35930840

[47] Park E-J, Han J-S, Park E-J et al. Repeated-oral dose toxicity of polyethylene microplastics and the possible implications on reproduction and development of the next generation. *Toxicol Lett* 2020;324:75–85. 10.1016/j.toxlet.2020.01.008 31954868

[48] Meyerstein D . Re-examining Fenton and Fenton-like reactions. *Nat Rev Chem* 2021;5:595–7. 10.1038/s41570-021-00310-4 37118415

[49] Derr R, Moelijker N, Hendriks G et al. A tiered approach to investigate the inhalation toxicity of cobalt substances. Tier 2 b: reactive cobalt substances induce oxidative stress in ToxTracker and activate hypoxia target genes. *Regul Toxicol Pharmacol* 2022;129:105120. 10.1016/j.yrtph.2022.105120 35038485

[50] Burgum MJ, Ulrich C, Partosa N et al. Adapting the in vitro micronucleus assay (OECD Test Guideline No. 487) for testing of manufactured nanomaterials: recommendations for best practices. *Mutagenesis* 2024;39:205–17. 10.1093/mutage/geae010 38502821 PMC11040148

