# Supplementary material for: Understanding the cellular uptake and genotoxic potential of industrial relevant nanomaterials utilizing electron microscopy and the ToxTracker assay in vitro
Source: Mutagenesis. 2025 Jul 17;40(4):550–9. doi: 10.1093/mutage/geaf013 (PMC12534216; doi:10.1093/mutage/geaf013)
Supplement: Supplementary_Figs_geaf013 [file supplementary_figs_geaf013.pdf]

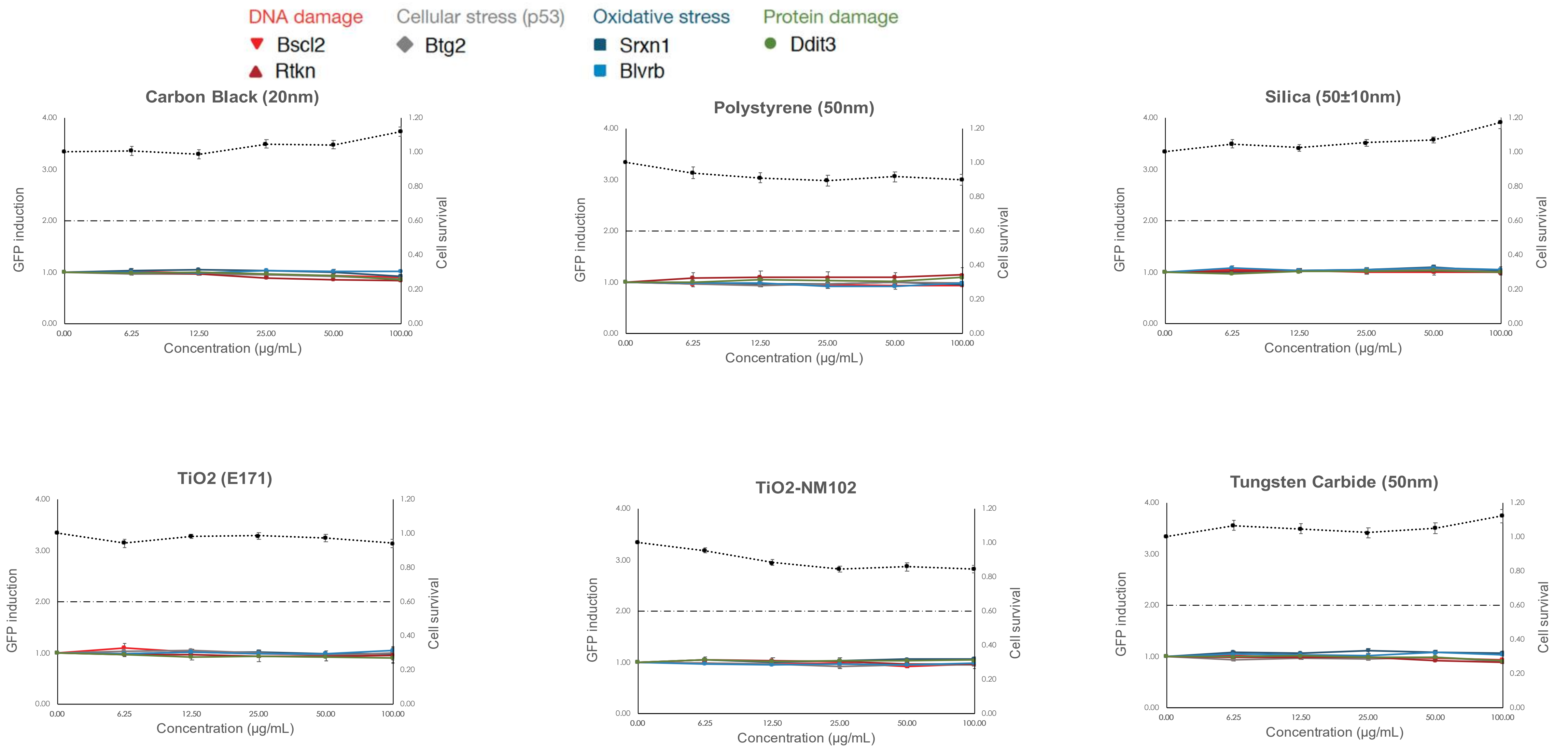

Supplementary figure 1 – ToxTracker analysis of Carbon Black, Polystyrene Silica, TiO_2 (E171), TiO_2-NM102 and, Tungsten Carbide showing no change in reporter with increasing test concentrations.

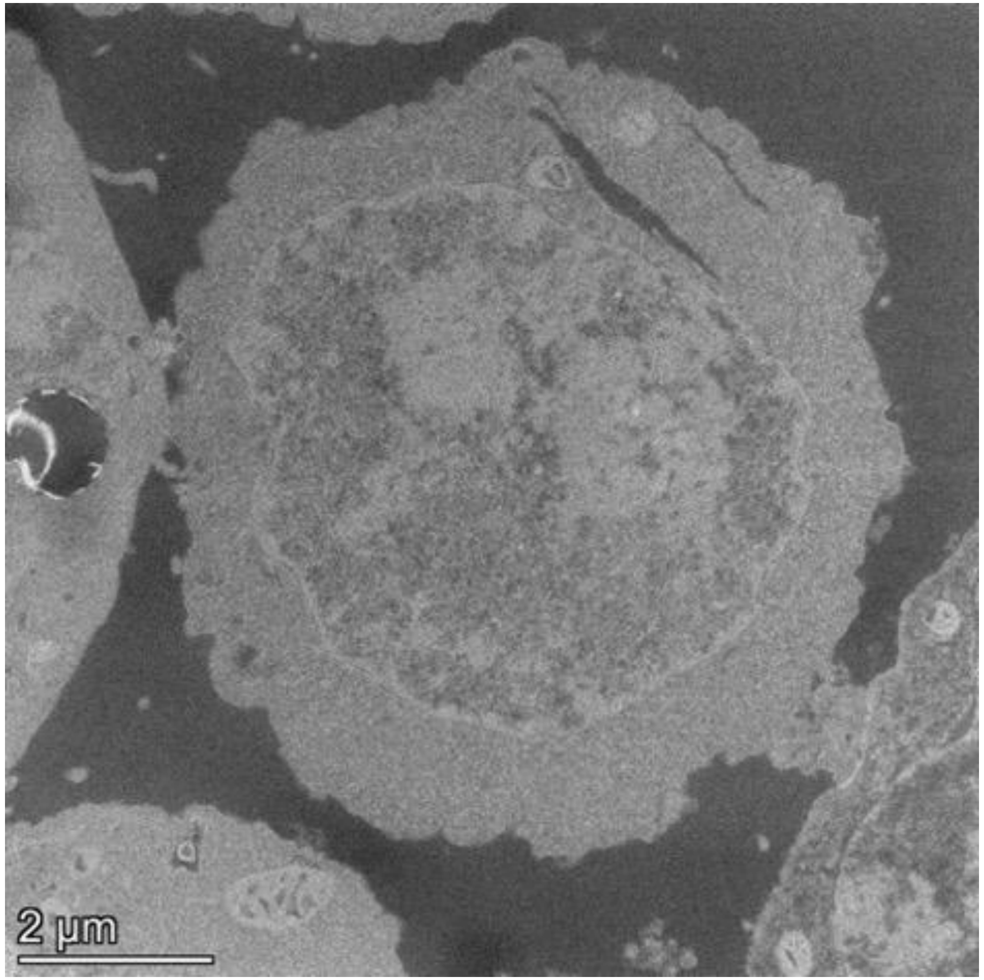

Supplementary figure 2 – Representative TEM image of mES cell that has not taken up any test material.
